# Association Between Reduced Daily Protein Intake and Sarcopenic Obesity in Men Living with HIV: A New Screening Tool

**DOI:** 10.3390/nu17193042

**Published:** 2025-09-24

**Authors:** Carla Greco, Leila Itani, Jovana Milic, Michela Belli, Silvia Gabriele, Mariagrazia Conti, Filippo Valoriani, Giovanni Guaraldi, Vincenzo Rochira, Marwan El Ghoch

**Affiliations:** 1 Endocrinology Unit, Department of Medical Specialties, Azienda Ospedaliero-Universitaria di Modena, 41126 Modena, Italy; carlagreco@unimore.it (C.G.); rochira.vincenzo@unimore.it (V.R.); 2 Department of Nutrition and Dietetics, Faculty of Health Sciences, Beirut Arab University, Riad El Solh, Beirut P.O. Box 11-5020, Lebanon; l.itani@bauedu.lb; 3 Department of Surgical, Medical, Dental and Morphological Sciences, University of Modena and Reggio Emilia, 41124 Modena, Italy; jovana.milic@gmail.com (J.M.); michela.belli13@gmail.com (M.B.); giovanni.guaraldi@unimore.it (G.G.); 4 Metabolic Diseases and Clinical Nutrition Unit, Department of Medical Specialties, Azienda Ospedaliero-Universitaria di Modena, 41126 Modena, Italy; gabriele.silvia@aou.mo.it (S.G.); conti.mariagrazia@aou.mo.it (M.C.); valoriani.filippo@aou.mo.it (F.V.); 5 Endocrinology Unit, Department of Biomedical, Metabolic and Neural Sciences, University of Modena and Reggio Emilia, 41126 Modena, Italy; 6 Center for the Study of Metabolism, Body Composition and Lifestyle, Department of Biomedical, Metabolic and Neural Sciences, University of Modena and Reggio Emilia, 41125 Modena, Italy

**Keywords:** adiposity, handgrip, BMI, body composition, body fat, overweight, muscle

## Abstract

**Background and Aim:** Sarcopenic obesity (SO) is a phenotype characterized by increased body fat combined with reduced muscle mass and strength. SO is prevalent among people living with HIV, especially in men (MLWH); however, the link between this phenotype and diet is still unclear in this population. For this reason, in this study, we aim to examine potential associations between self-reported macronutrient intake and SO in MLWH, and, eventually, to evaluate the diagnostic accuracy of a simple nutritional marker for screening SO. **Methods:** A total of 216 MLWH were selected from a large cohort who completed a total body composition measurement by dual-energy X-ray absorptiometry (DXA), muscle strength assessment by handgrip test, and nutritional recording by 24 h recall interview. The sample was categorized into SO (*n* = 45), non-SO (NSO) (n = 33), and non-sarcopenic non-obesity (NSNO) (*n* = 138). Logistic regression analysis was performed to determine the associations between different macronutrients and SO after adjusting for confounders. Receiver operating characteristic (ROC) curve analysis was used to identify discriminating cut-off points of the determined macronutrient intake to screen for SO. **Results:** The MLWH with SO while compared to NSO and NSNO, were of an older age and had a higher BMI, but with a lower total caloric and protein intake. However, adjusted logistic regression showed that only protein intake (g/kg/day) (OR = 0.017; 95%CI: 0.003–0.094, *p* < 0.05) and age (OR = 1.051; 95%CI: 1.011–1.093, *p* < 0.05) were significantly associated with SO. The age-adjusted ROC analysis identified the 0.98 g/kg/day of protein intake (AUC = 0.8149; *p* < 0.0001; sensitivity = 71%; specificity = 70%) as a cut-off point to screen for SO in the MLWH. **Conclusions:** We identified a new cut-off point of daily protein intake able to screen for SO in MLWH, and its use can be implemented in clinical settings.

## 1. Introduction

The human immunodeficiency virus (HIV) infection remains one of the most serious public health challenges [1]. In 2023, on a global scale, around 40 million people were living with HIV (PLWH) [1]. Despite the decline in new infections since the late 1990s and early 2000s [2], and the impressive advancements in the clinical management of this population [3], the PLWH still remain at higher risk of non-communicable comorbidities, including cardiovascular, metabolic, and kidney disease [4]. They are also more affected by psychological and psychiatric conditions such as depression, psychosis, substance use disorder, and post-traumatic stress disorder [5] leading to suboptimal health-related quality of life (HRQoL) [6]. These complications may result from chronic HIV-related inflammation, long-term antiretroviral therapy (ART), or the infection itself [7].

Sarcopenic obesity (SO) is considered a relatively new clinical phenotype, which has emerged in the past two decades [8,9]. It is characterized by an increase in fat deposition with abnormal distribution, combined with a reduction in muscle mass and strength [10]. This phenotype has garnered much interest in several clinical settings [11], and has been the subject of in-depth investigation, revealing its association with a higher risk of medical (i.e., type 2 diabetes, hypertension, dyslipidemia, etc.) [12] and psychosocial (i.e., depression, anxiety, and HRQoL) comorbidities [13] in several clinical populations of different ethnic, age, and gender groups.

In this direction, and despite the paucity of studies on SO in PLWH, the recent literature has claimed that SO appears to be a prevalent condition in PLWH [14,15], especially in MLWH, as their co-existence is more associated with unfavorable clinical outcomes, in terms of clinical comorbidities (i.e., cardiovascular diseases, metabolic syndrome, and osteoporosis) [15], major frailty [16], and poorer HIV-related outcomes (i.e., lower frequency of CD4+ T cells) [17]. Chronic low-grade inflammation is the common denominator between HIV-infection and SO that may lead to a bi-directional interaction between the two conditions [18,19,20]. For this reason, it is vital to identify this condition (i.e., SO) promptly to prevent and manage related comorbidities in PLWH. However, and according to recent consensus by the European Society for Clinical Nutrition and Metabolism (ESPEN) and the European Association for the Study of Obesity (EASO) [10], the diagnosis of SO should rely on a multi-dimensional evaluation based an accurate measurement of body fat (BF), appendicular lean mass (ALM), and muscle strength, all of which require time, sophisticated machines, and well-trained healthcare professionals (i.e., technicians, physiotherapists, etc.). Therefore, the identification of a simple-to-use tool to screen for SO has become of clinical relevance in this population.

On a general scale, SO was found to be linked to several dietary and nutritional factors, such as diet quality, appropriate intake of calories and protein, and consumption of antioxidant nutrients, vegetables, fruits, etc. [21]. However, to date, there is no clear and updated consensus regarding the nutritional requirements in PLWH [22,23]. Moreover, to the best of our knowledge, so far, no study has been conducted revealing any potential association between nutritional factors and SO in PLWH.

Based on these considerations, the aim of the current study was to assess potential associations between nutritional factors and SO in MLWH. A secondary objective was to identify a simple, easy-to-use screening tool to detect individuals at higher risk of SO in this population, based on a simplified dietary recall that includes macronutrient daily intake.

## 2. Materials and Methods

This is an observational retrospective cross-sectional study. Participants were selected from a cohort of 5317 PLWH consecutively admitted and regularly attending the Modena HIV Metabolic Clinic (MHMC) between May 2001 and May 2025. Participants were considered eligible if they had a complete dietary dataset (i.e., daily caloric and macronutrient intake), and a total of 830 participants satisfied eligibility. The inclusion criteria were as follows: (i) having an age ≥20 years; (ii) being male; (iii) having performed a muscle strength assessment, and (iv) having performed a total and segmental body composition measurement by means of dual-energy X-ray absorptiometry (DXA). Participants were excluded if they were as follows: (i) children or adolescents aged ≤19 years [24]; or (ii) female. A total of 216 MLWH were included, and the sample was categorized into SO (*n* = 45), non-SO (NSO; obesity without sarcopenia) (*n* = 33), and non-sarcopenic non-obesity (NSNO) (*n* = 138).

The research adhered to the Declaration of Helsinki and was approved on 10 April 2024, by the Institutional Review Board (IRB) of Area Vasta Emilia Nord (Protocol n. 0010488/24). All participants’ personal data were treated according to European privacy laws, and informed written consent was obtained.

### 2.1. Demographics and Clinical Status

Variables regarding demographical and clinical status related to HIV infection were obtained by interview and chart review, taking into account the following: age, gender, time since HIV diagnosis, total cumulative exposure to ART, undetectable HIV viral load, lymphocyte CD4+ count as an absolute value and as a percentage, and the ratio CD4 to CD8.

### 2.2. Body Weight and Height

Body weight and height were measured by a dietician involved in the study after at least a five-hour fast before breakfast, wearing light clothes and no shoes, using an analogical weighing scale and a stadiometer. The BMI was then calculated according to the standard formula of body weight measured in kilograms, divided by the square of the height in meters [25]:

BMI (kg/m^2^) = body weight (kg) ÷ height^2^ (m).

### 2.3. Body Composition

Body composition was measured by dual-energy X-ray absorptiometry (DXA) (DXA, Hologic-QDR-2000 densitometer, Inc., Waltham, MA, USA) according to standardized procedures [26]. Precision error rates for the QDR 2000 were 3% for the measurement of BF and 1.5% for LM. The DXA machine underwent calibration daily to ensure accurate measurements using an anthropometric phantom. Individuals were asked to abstain from any form of physical activity for the 24 h prior to the measurement. Before the test, and prior to being positioned on the DXA table, they were asked to remove all clothing, including shoes, socks, and metal items except for undergarments. Scans were performed with the individuals in a supine position. The entire body was scanned, beginning from the top of the head and moving in a rectilinear pattern down the body to the feet. The average measurement time was 20 min. In this study, the following variables were considered:

Body fat (BF) = total body fat expressed in kg.

BF% (BF as a percentage of the total mass) = (BF ÷ body weight) × 100.

Appendicular lean mass (ALM) = total lean in arms and legs with bone  excluded, expressed in kg).

### 2.4. Definition of Sarcopenic Obesity (SO)

The definition of SO was established in adherence to the consensus statement released by ESPEN and EASO [10]. The diagnostic procedures initially included assessment of skeletal muscle function, followed by assessment of body composition, where the presence of reduced muscle strength, excess adiposity (i.e., BF%), and low skeletal muscle mass was all necessary to confirm the diagnosis of SO as follows:

- Muscle strength: muscle strength was assessed through handgrip strength (HGS) measured by Jamar dynamometer, asking the patient to squeeze the dynamometer tightly with maximum force, then to release it. Measurements were made three times in each hand, and the average of the three measurements was used for analysis. Low muscle strength was defined according to BMI-adjusted cut-off points suitable for PLWH [27]:
  ➢ For males: HGS/BMI < 1.05.
- Low muscle mass was established according to the ratio of the appendicular lean mass divided by weight (ALM/W) [10]:
  ➢ For males ALM/W < 28.27%.
- Obesity was defined according to adiposity based on age- and male-specific BF% cut-offs [28]:
  ➢ 18–39 years: BF% ≥ 26%.
  ➢ 40–59 years: BF% ≥ 29%.
  ➢ 60–98 years: BF ≥ 31%.

### 2.5. Food Intake

The food intake was collected by a dietitian using a 24 h recall interview, considering the following variables:

- Total caloric intake (kcal/day): the total calories from all food and beverages consumed in 24 h expressed as kcal/day.
- CHO g/day: the daily total quantity of carbohydrate consumed in 24 h expressed in grams.
- CHO% of total kcal/day: the proportion of total carbohydrate out of total calories consumed in 24 h expressed as a percentage.
- Complex CHO g/day: the daily total quantity of complex carbohydrate consumed in 24 h expressed in grams.
- Complex CHO% of total kcal/day: the proportion of complex carbohydrate out of total calories consumed in 24 h expressed as a percentage.
- Simple CHO g/day: the daily total quantity of simple carbohydrate consumed in 24 h expressed in grams.
- Simple CHO% of total kcal/day: the proportion of simple carbohydrate out of total calories consumed in 24 h expressed as a percentage.
- Fiber g/1000 kcal: the quantity of total fiber in 1000 calories in the 24 h expressed in grams per 1000 kcal.
- Fiber daily intake (fiber < 28 g/day or fiber ≥ 28 g/day): participants were classified according to their daily total fiber intake, above or below the cut-off point of 28 g/day.
- Total protein intake (g/day): the daily total quantity of proteins consumed in 24 h expressed in grams.
- Protein (g/kg/day): the daily quantity of total proteins consumed in 24 h expressed in grams per kilogram of body weight per day.
- Protein% of total kcal/day: the proportion of total protein out of total calories consumed in 24 h expressed as a percentage.
- Total fat g/day: the daily total quantity of fat consumed in 24 h expressed in grams.
- Fat% of total kcal/day: the proportion of total fat out of total calories consumed in 24 h expressed as a percentage.
- PUFA g/day: the daily total quantity of polyunsaturated fat consumed in 24 h expressed in grams.
- PUFA% of total kcal/day: the proportion of polyunsaturated fat out of total calories consumed in 24 h expressed as a percentage.
- MUFA g/day: the daily total quantity of monounsaturated fat consumed in 24 h expressed in grams.
- MUFA% of total kcal/day: the proportion of monounsaturated fat out of total calories consumed in 24 h expressed as a percentage.
- Saturated fat g/day: the daily total quantity of saturated fat consumed in 24 h expressed in grams.
- Saturated fat % of total kcal/day: the proportion of saturated fat out of total calories consumed in 24 h expressed as a percentage.
- Dietary cholesterol (mg/day): the daily total quantity of cholesterol consumed in 24 h expressed in milligrams per day.

### 2.6. Statistical Analysis

Normality was assessed using the Shapiro–Wilk test, and significant results (*p* < 0.05) indicated the non-normality of the variable distribution [29,30,31]. Accordingly, means and standard deviations or medians and interquartile ranges were reported for normal or non-normal variables, respectively. For comparison between groups, univariate analysis using the ANOVA test or the nonparametric Kruskal–Wallis test with multiple comparisons was used. Multiple comparisons were adjusted for Bonferroni correction, and an adjusted *p* value less than 0.05 was considered significant. Categorical variables were reported as frequencies and percentages, and a chi-squared test for independence was used to assess the association between categorical variables. A chi-squared test was conducted after meeting the criteria that the frequency values of expected cells are≥5 in at least 80% of the cells, with no cells having expected frequency less than 1 [32].

Logistic regression analysis was conducted to identify the main determinant of SO. Based on univariate results (an ANOVA or Kruskal–Wallis test), variables with *p* values less than 0.05 were selected initially for inclusion in the logistic regression model. Next, the purposeful selection described by Bursac and colleagues was employed, and accordingly, a simple logistic model for every variable selected initially (*p* < 0.05) was constructed [33]. All variables with *p* values for the Wald test ≤0.25 in the simple model were all included in the final multivariate model. Moreover, in the final model, those variables with *p* values > 0.1 would be kept, given that removal would cause a more than 15–20% change in the coefficient of the other variables [33], except for total energy intake, which was kept as a known confounder from the literature [34]. Accordingly, the final model included age, energy, and fiber intake in addition to the standardized protein intake. The final logistic regression model fit was examined based on the Hosmer–Lemeshow test (*p* > 0.05), model accuracy, and model discriminative ability as measured by AUC, with an AUC of 0.5 indicating no discrimination, 0.5–0.7 poor discrimination, 0.7–0.8 acceptable discrimination, 0.8–0.9 excellent discrimination, and ≥0.9 outstanding discrimination [35,36]. To test multicollinearity among the predictors included in the model, the pairwise Pearson’s correlation and VIF from a linear regression model with the predictors were used. Predictors with a paired ρ < 0.7 and VIF less than 2.5 from the linear regression model and an SE < 5 for each variable in the logistic model were considered to pose to significant threat for multicollinearity [37,38,39].

To evaluate the diagnostic performance of the determinant of SO (identified by the logistic regression model) in detecting SO status, a classification analysis was performed by calculating sensitivity and specificity as well as the area under the curve (AUC) of the receiver operating characteristic curve (ROC). The criterion value of the determinant with maximum sensitivity and specificity was selected for the cut-off point. An AUC between 0.7 and 0.8 indicated the criterion value had acceptable discriminating power, an AUC between 0.8 and 0.9 indicated the criterion value had an excellent discriminating ability, and more than 0.9 was considered outstanding [40,41]. All values were considered significant at *p* < 0.05. The Number Cruncher Statistical Systems (NCSS) (ver. 23.0.2) (NCSS, 2023) package [42], and the statistical package for social sciences SPSS (ver.26) for statistical analysis, and RStudio (ver. 2025.05.1.513) [43] were used to perform the age-adjusted ROC analysis.

## 3. Results

The study included 216 MLWH with a mean age of 49.98 ± 10.76 years and a median BMI of 23.92 kg/m^2^ (IQR: 21.96–26.79 kg/m^2^). Based on the WHO BMI classification system, 36.6% were classified as having overweight or obesity. The median BF was 19.84 kg (IQR: 15.77–24.78 kg) with a median BF% of 26.88% (IQR: 23.09–30.88). Using Gallagher’s criteria classification system for obesity definition based on level of adiposity (i.e., BF%) [28], 36.1% had obesity, and 41.2% were overweight. The median total ALM was 21.65 kg (IQR: 20.02–23.84 kg), and the median proportion of ALM from total body weight was 29.65% (IQR: 28.46–31.27%). The mean HGS was 39.96 ± 9.05 kg, and the median HGS/BMI was 1.59 (IQR: 1.17–1.87) (Table 1). The median duration since HIV diagnosis was 12.5 years (IQR: 5.25–22.69 years), and the median duration on ART treatment was 11 years (IQR: 5.00–21.00 years). Relatively, the immune cell counts reflected good immunological status.

Among the MLWH included in the study, 45 (20.8%) had SO, 33 (15.3%) had NSO, and the remaining 138 (63.9%) patients had NSNO. The MLWH who had SO were significantly older (56.02 ± 10.30 years) than those in the NSO (43.76 ± 10.54 years) and NSNO (49.50 ± 10.00 years) groups. The MLWH demonstrated a significantly decreasing BMI across SO categories with those with SO having the highest median BMI (30.85 kg/m^2^ (IQR: 27.75–34.76 kg/m^2^)) followed by those with NSO (24.34 kg/m^2^ (IQR: 22.85–26.24 kg/m^2^)), with the least median BMI being among those in the NSNO group (23.06 kg/m^2^ (IQR: 21.41–24.24 kg/m^2^)) (*p* < 0.05). The prevalence of obesity and overweight as assessed by BMI was highest among the MLWH having SO (91.1% vs. 42.4% and 17.4%) (χ^2^: 80.075; *p* < 0.05). BF and BF% followed a similar trend, with the highest median BF observed among those with SO (32.27 kg (IQR: 27.33–39.82 kg)), followed by those with NSO (22.70 kg (IQR: 21.72–25.57 kg) and least among those with NSNO (17.05 kg (IQR: 13.88–19.95 kg)) (*p* < 0.05). The median BF% was highest among the MLWH who have SO (37.66% (IQR: 34.62–40.09%)), followed by those with NSO (29.66% (IQR: 28.40–31.06%)), with the least observed being among those with NSNO (24.46% (IQR: 21.47–26.82%)) (*p* < 0.05). While the median ALM did not differ across the SO, NSO, and NSNO categories (21.26 kg (IQR: 18.97–24.99 kg) vs. 23.52 kg (IQR: 21.31–24.85 kg) vs. 21.53 kg (IQR: 20.17–23.60 kg), the median ALM/W% followed a significantly increasing trend across categories, unlike BMI or BF and BF%. The median ALM/W% was least among the MLWH who have SO (24.24% (IQR: 22.55–25.94%)), followed by those with NSO (28.92% (IQR: 28.69–30.16%)), with the highest being among those with NSNO (30.60% (IQR: 29.52–32.11%)) (*p* < 0.05). The mean HGS was significantly lower among the MLWH who have SO (26.02 ± 5.85 kg) as compared to the other two categories, NSO (40.08 ± 8.32 kg) or NSNO (39.79 ± 7.20 kg). HGS/BMI followed the same increasing trend with those having SO demonstrating the least median HGS/BMI (0.84 (IQR: 0.75–0.95)) compared to those with NSO (1.65 (IQR: 1.43–1.83)) or NSNO (1.70 (IQR: 1.52–1.96)) (*p* < 0.05) (Table 1). Blood parameters related to HIV did not vary across categories of SO, indicating well-controlled viral status (Table 1).

The intake of macronutrients among MLWH is presented as an absolute value and adjusted to nutrient density (% kcal for macronutrients and g per 1000 kcal for fiber). Overall, the MLWH consumed close to 2000 kcal/day with a median intake of 1918 kcal (IQR: 1650–2211 kcal). The median CHO intake was 234.00 g/day (IQR: 183.75–280.50 g/day), which constituted almost 50% of the total energy intake with a median of 49.27% (IQR: 42.71–53.61%). Simple CHO median intake was 68.00 g/day (50.00–86.00 g/day), constituting around 14% of total energy with a median of 14.31% (10.63–17.95%). Fiber intake was low, reaching a median of 9.98 g/1000 kcal (7.50–12.80 g/1000 kcal), with almost 80% (79.2%) consuming less than 28 g fiber per day. Total protein intake reached a median of 80.50 g/day (IQR: 66.75–98.00 g/day), approaching almost 20% of total daily energy with a median of 16.77% (IQR: 14.26–19.47%). The median protein intake adjusted by body weight was 1.05 g/kg (IQR: 0.86–1.27 g/kg). For dietary fat, the median intake was 70.00 g/day (IQR: 57.00–82.00 g/day), which constituted one-third of total energy intake with a median of 32.77% (IQR: 28.76–37.01%). MUFA was the most consumed fat subtype (median: 15.52% (IQR: 13.55–18.42%)), followed by saturated fat (median: 9.10% (IQR: 7.24–11.25%)) and PUFA (median: 4.00% (IQR: 3.21–5.40%)). Dietary cholesterol intake did not exceed 300 mg, with a median intake of 190.00 mg/day (IQR: 133.00–250.00 mg/day) (Table 2).

The dietary intake across categories of SO varied only with respect to total energy intake (kcal/day), absolute protein gram intake per day, and standardized protein intake as grams per kg bodyweight, but not as a percentage of total energy or fiber intake. Daily energy intake was least among those with SO (median: 1850 kcal (IQR: 1410–2100 kcal)) compared to those with NSO (median: 2050 kcal (IQR: 1708–2330 kcal)) and NSNO (median: 1972 kcal (IQR: 1730–2321 kcal)). Those with SO were more likely to consume <28 g fiber per day (93.3% compared to those with NSO (66.7%) and NSNO (77.5%) (χ^2^: 6.917; *p* < 0.05). Remarkably, the standardized protein intake per kg body weight demonstrated a significantly increasing trend across categories, with those with SO having the least protein intake per kg with a median of 0.87 g/kg (IQR: 0.63–1.01 g/kg) compared to NSO (median: 1.08 (IQR: 0.87–1.28)) or NSNO (median: 1.17 (IQR: 0.99–1.51)). Other dietary intake variables did not vary across categories of SO (Table 2).

Based on the above univariate analysis, the only variables that were significantly associated with SO were standardized protein intake per kg of body weight (g/kg), fiber intake below 28 g per day, energy intake, and age (*p* < 0.05). Simple logistic regression models for each of the four variables alone fulfilled the criteria of a *p* ≤ 0.25 for the Wald test. In the final model, energy intake was retained in the model because it is a known confounder from the literature [34], despite the *p* value being >0.1. After adjustment for age, energy, and fiber intake, the final model showed that the odds of SO decreased by 9.8% for each 0.1 g/kg increase in standardized protein intake per kg of body weight (gram protein/kg) (OR: 0.017; CI: 0.003–0.094, *p* < 0.0001) (Table 3). The logistic regression model achieved an AUC of 0.83, indicating excellent discrimination ability.

**Figure 1:**
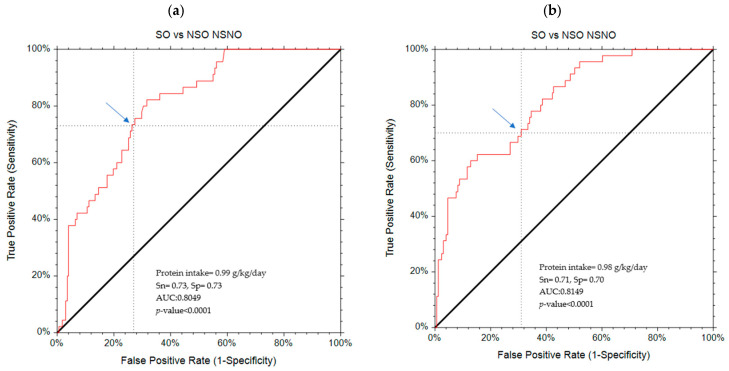
ROC curves for unadjusted and adjusted analysis. (**a**) Unadjusted ROC curve; (**b**) Age-adjusted ROC curve. The blue arrows mark the sensitivity and specificity of the cut-off point on the ROC curve.

The ROC analysis showed that the most appropriate protein gram per kg cut-off point for identifying SO was 0.99 g/kg/day. This cut-off point achieved a good sensitivity (73%) and specificity (73%), indicating a relatively low chance for false positives or false negatives. The AUC of 0.8 depicts an excellent discriminating ability of the cut-off point, which has an 80% chance of detecting SO. Considering an age-adjusted model only, a cut-off point of 0.98 g protein kg was identified with a sensitivity and specificity for predicted protein intake per kg of 71% and 70%, respectively, indicating a relatively low chance for false positives or false negatives. The AUC of 0.8 depicts a very good discriminating ability of the cut-off point, which has an 80% chance of detecting SO (Table 4 and Figure 1).

## 4. Discussion

The present study aimed to assess the potential association between macronutrient intake and SO in MLWH, and, if possible, to evaluate whether macronutrients could serve as a simple, easy-to-use screening tool for SO in this population.

### 4.1. Findings and Concordance with Previous Studies

Firstly, we found a strong and independent association between daily protein intake (g/kg/day) and SO in MLWH. Although no previous study has reported this specific association (i.e., SO), our finding is consistent with the previous literature showing that low daily protein intake is related to sarcopenia in PLWH [44]. However, it contrasts with other studies that found no association between reduced protein intake and higher prevalence of sarcopenia in PLWH [45]. Moreover, our study went beyond simply reporting the association between protein intake and SO in MLWH. We also identified a new cut-off point of 0.99 g/kg/day for protein intake as a potential screening value for SO in this population. The concept of using dietary macronutrient intake to predict SO is novel and of particular clinical interest [46], especially because the diagnosis of SO needs a complex procedure, through a multi-dimensional and time-consuming evaluation that requires sophisticated instruments (i.e., DXA) and well-trained professionals. The identification of an easy-to-use tool, such as a nutritional recall that can screen individuals for a higher risk of SO, is of clinical relevance, since it saves time and manpower resources as well as medical expense, a known burden for healthcare systems [47]. To the best of our knowledge, this is the first study to establish such a screening tool. However, the observed association between daily protein intake and SO does not imply a direct causal relationship between the two in MLWH, and further research with longitudinal assessments is needed.

### 4.2. Strengths and Limitations

Our study has several strengths. To the best of our knowledge, it is one of the few evaluations to establish an easy-to-use tool based on daily protein intake per unit of body weight to screen for SO in a “real-world” MLWH population setting in Italy. Second, our study strictly adhered to the recently updated definition of SO according to the consensus by EASO and ESPEN [10]. Moreover, body composition was measured using DXA, which is known to also exhibit a high level of precision in PLWH [48]. However, our investigation also had some limitations. Most importantly, the data were obtained from one treatment center in Italy and only from males of a small sample size; therefore, external validation in others from a larger sample, as well as in women, is necessary, as data regarding SO components and dietary intake were not sufficiently available in our initial cohort. Therefore, our findings cannot be extended to other populations and should be interpreted with caution [49]. Secondly, our nutritional data were limited to macronutrient intake, as micronutrient data were lacking in our study; therefore, we were not able to assess their association with SO in MLWH. Thirdly, several lifestyle factors such as physical activity levels [50], sleeping [51], and smoking habits [52] were lacking, factors known to influence body composition compartments (i.e., BF and LM). Finally, our study was of a cross-sectional design [53]; therefore, it was unable to detect a relationship between protein intake as a cause of SO in MLWH, which usually requires longitudinal assessment [54], to understand the beneficial effect of increasing dietary intake of protein/day in these patients, and whether this can prevent SO onset or/and can promote its amelioration and arrest its progression [55].

### 4.3. Clinical Implications and New Directions for Future Research

If confirmed, our findings have certain clinical implications. Firstly, healthcare services dealing with MLWH in Italy may use our results as at least preliminary evidence for considering the importance of the dietary recall assessment, especially when related to daily protein intake, as a quantity roughly inferior than 1 g/kg/day in a MLWH should be highly indicative of SO and may serve as a red flag for clinicians lacking access to DXA or handgrip dynamometers, and this should also be openly discussed with the patient. Furthermore, none of the HIV-related variables that we took into consideration were associated with SO, implying that all MLWH, regardless of HIV duration or viro-immunological status, should be considered at risk and, therefore, screened for SO.

Future research in several directions is still needed on the topic. Firstly, more studies should be conducted on SO, in terms of onset, for a better understanding of the pathophysiology of this phenotype in PLWH, as well as its progression and treatment in this population. Secondly, based on the evidence, new updated nutritional guidelines are strongly needed, taking into account SO in PLWH, since the currently available guidelines from the World Health Organization (WHO) were released more than two decades ago [56]. Thirdly, and consistent with our findings, there is a need to identify other simple tools that can be useful for SO screening among both men and women across different ages and ethnic backgrounds. Moreover, other works should extend the aim of our analysis to other European countries (i.e., Central, Eastern, Northern, Southern, and Western Europe) as well as on a global scale (Asia, the Middle and Far East, and South and North America).

## 5. Conclusions

Our study provides evidence that there is a link between nutritional intake, namely daily protein intake, and SO in MLWH that can be easily screened for via daily protein intake recall, which is a simple assessment that can be implemented in several healthcare settings dealing with PLWH; however, before implementation, more investigation is still needed for other PLWH groups (i.e., females) to be conducted to confirm and generalize our findings.

## Figures and Tables

**Table 1:** Characteristics of the MLWH across three categories of SO, NSO, and NSNO (*n* = 216).

	SO (*n* = 45)	NSO (*n* = 33)	NSNO (*n* = 138)	Total (*n* = 216)	Significance
Anthropometric parameters					
Age (years)	56.02 (10.30) ^a^	43.76 (10.54) ^b^	49.50 (10.00) ^c^	49.98 (10.76)	<0.05 ^£^
Weight (kg)	87.00 (76.75–102.50) ^a^	77.00 (73.50–83.00) ^a^	70.00 (64.00–75.50) ^b^	74.00 (66.70–81.93)	<0.05 ^¥^
Height (cm)	169.47 (6.76) ^a^	177.27 (6.02) ^b^	174.93 (6.17) ^b^	174.15 (6.74)	<0.05 ^£^
BMI (kg/m^2^)	30.85 (27.75–34.76) ^a^	24.34 (22.85–26.24) ^b^	23.06 (21.41–24.24) ^c^	23.92 (21.96–26.79)	<0.05 ^¥^
					χ^2^: 80.075; *p* < 0.001
BMI < 25.0 kg/m^2^	4 (8.9)	19 (57.6)	114 (82.6)	137 (63.4)	
BMI ≥ 25.0 kg/m^2^	41 (91.1)	14 (42.4)	24 (17.4)	79 (36.6)	
BF (kg)	32.27 (27.33–39.82) ^a^	22.70 (21.72–25.57) ^b^	17.05 (13.88–19.95) ^c^	19.84 (15.77–24.78)	<0.05 ^¥^
BF (%)	37.66 (34.62–40.09) ^a^	29.66 (28.40–31.06) ^b^	24.46 (21.47–26.82) ^c^	26.88 (23.09–30.88)	<0.05 ^¥^
ALM (kg)	21.26 (18.97–24.99)	23.52 (21.31–24.85)	21.53 (20.17–23.60)	21.65 (20.02–23.84)	>0.05 ^¥^
ALM/weight (%)	24.24 (22.55–25.94) ^a^	28.92 (28.69–30.16) ^b^	30.60 (29.52–32.11) ^c^	29.65 (28.46–31.27)	<0.05 ^¥^
HGS (kg)	26.02 (5.85) ^a^	40.08 (8.32) ^b^	39.79 (7.20) ^b^	39.96 (9.05)	<0.05 ^£^
HGS/BMI	0.84 (0.75–0.95) ^a^	1.65 (1.43–1.83) ^b^	1.70 (1.52–1.96) ^b^	1.59 (1.17–1.87)	<0.05 ^¥^
HIV-related variables					
Time since HIV diagnosis (years)	14.17 (6.60–22.81)	11.54 (5.29–20.48)	12.13 (4.96–23.85)	12.50 (5.25–22.69)	>0.05 ^¥^
ART exposure (years)	13.00 (6.50–21.00)	11.00 (5.00–19.00)	9.00 (4.00–21.50)	11.00 (5.00–21.00)	>0.05 ^¥^
HIV viral load (copies/mL)	24.00 (20.00–40.00)	40.00 (20.00–40.00)	38.00 (20.00–40.00)	38.00 (20.00–40.00)	>0.05 ^¥^
					χ^2^: 2.271; *p* = 0.321
Undetectable (<50)	44 (97.8)	33 (100)	131 (94.9)	208 (96.3)	
Detectable (≥50)	1 (2.2)	0 (0.0)	7 (5.1)	8 (3.7)	
Lymphocytes CD4 absolute value (cells/μL)	669.00 (316.60)	730.39 (255.38)	763.21 (657.55)	738.27 (553.90)	>0.05 ^£^
Lymphocytes CD4 (%)	30.59 (9.82)	35.26 (7.92)	33.89 (9.33)	33.39 (9.33)	>0.05 ^£^
CD4:CD8 ratio	0.84 (0.49)	1.71 (3.86)	1.12 (1.40)	1.15 (1.90)	>0.05 ^£^

Values are either means (SD) or medians (IQR) for continuous variables and *n* (%) for categorical variables; ^¥^ Results for Kruskal–Wallis test with multiple comparisons, significance is considered based on *p* values for Bonferroni correction for multiple comparisons; ^£^ Results for ANOVA test with multiple comparisons, significance is considered based on *p*-values for Bonferroni Correction for multiple comparisons; ^a,b,c^ values wih different superscripts are significantly different at *p* < 0.05; SO = sarcopenic obesity; NSO = non sarcopenic obesity; NSNO = non sarcopenic non obesity; BMI = body mass index; BF = body fat; BF% = body fat percentage; ALM = appendicular lean mass; HGS = handgrip strength; HIV = human immunodeficiency virus; ART = Antiretroviral Therapy.

**Table 2:** Dietary Intake among the MLWH across three categories of SO, NSO, and NSNO (*n* = 216).

Dietary Variable (*n* = 216)	SO (*n* = 45)	NSO (*n* = 33)	NSNO (*n* = 138)	Total (*n* = 216)	Significance ^¥^
Caloric intake (kcal/day)	1850.00 (1410.00–2100.00) ^a^	2050.00 (1707.50–2329.50) ^a,b^	1972.00 (1730.00–2321.00) ^b^	1917.50 (1650.00–2211.25)	<0.05
Carbohydrate					
Total CHO (g/day)	218.00 (156.00–259.00)	242.00 (179.00–293.50)	248.50 (198.00–287.00)	234.00 (183.75–280.50)	>0.05
CHO% of total (kcal/day)	48.08 (41.17–54.05)	48.98 (43.93–52.22)	49.58 (43.53–54.84)	49.27 (42.71–53.61)	>0.05
Complex CHO (g/day)	153.50 (120.75–189.00)	160.00 (129.00–193.50)	171.50 (128.75–218.25)	160.00 (123.75–205.00)	>0.05
Complex CHO% of total kcal/day	33.87 (28.23–40.68)	33.72 (27.83–36.51)	33.56 (28.79–39.69)	33.80 (28.79–38.92)	>0.05
Simple CHO (g/day)	58.00 (34.50–81.50) ^a^	72.00 (52.50–108.50) ^a,b^	72.50 (57.75–86.75) ^b^	68.00 (50.00–86.00)	<0.05
Simple CHO% of total kcal/day	13.71 (8.25–18.02)	14.51 (12.97–17.26)	14.09 (11.16–17.94)	14.31 (10.63–17.95)	>0.05
Fibers (g/1000 kcal)	8.89 (6.88–10.96)	10.63 (7.78–13.12)	9.98 (7.78–12.74)	9.98 (7.50–12.80)	>0.05
Fibers daily intake ^†^					χ^2^:6.917; *p* < 0.009
Fibers < 28 (g/day)	42 (93.3)	22 (66.7)	107 (77.5)	171 (79.2)	
Fibers ≥ 28 (g/day)	3 (6.7)	11 (24.4)	31 (22.5)	45 (20.8)	
Protein					
Total Protein (g/day)	77.00 (57.00–88.00) ^a^	82.00 (69.50–95.50) ^a,b^	85.00 (66.75–104.25) ^b^	80.50 (66.75–98.00)	<0.05
Protein (g/kg/day)	0.87 (0.63–1.01) ^a^	1.08 (0.87–1.28) ^b^	1.17 (0.99–1.51) ^b^	1.05 (0.86–1.27)	<0.05
Protein (% of total kcal/day)	16.84 (14.30–18.67)	16.54 (14.41–18.79)	17.12 (14.72–19.81)	16.77 (14.26–19.47)	>0.05
Fat					
Total Fat (g/day)	70.00 (50.00–78.00)	73.00 (54.00–88.00)	71.00 (59.75–86.00)	70.00 (57.00–82.00)	>0.05
Fat% of total kcal/day	32.91 (29.17–37.39)	34.37 (28.58–37.69)	32.27 (28.44–36.97)	32.77 (28.76–37.01)	>0.05
PUFA (g/day)	8.00 (5.00–11.25)	9.00 (7.00–13.25)	9.00 (7.00–13.75)	9.00 (7.00–12.00)	>0.05
PUFA (% of total kcal/day)	4.02 (2.88–5.64)	3.82 (3.35–5.46)	4.08 (3.24–5.40)	4.00 (3.21–5.40)	>0.05
MUFA (g/day)	32.00 (25.75–37.00)	37.00 (30.00–44.00)	34.00 (29.00–40.00)	34.00 (29.00–39.50)	>0.05
MUFA (% of total kcal/day)	15.25 (13.48–19.24)	16.26 (13.19–20.06)	14.93 (12.97–17.84)	15.52 (13.55–18.42)	>0.05
Saturated fat (g/day)	19.50 (13.75–24.25)	16.00 (12.75–26.50)	20.50 (14.25–26.00)	19.00 (14.50–25.00)	>0.05
Saturated fat (% of total kcal/day)	9.51 (7.36–11.72)	7.86 (6.53–10.14)	9.17 (7.06–11.03)	9.10 (7.24–11.25)	>0.05
Dietary cholesterol (mg/day)	198.00 (141.00–264.50)	199.50 (147.00–260.50)	194.00 (129.25–268.75)	190.00 (133.00–250.00)	>0.05

Values are medians (IQR) for continuous variables and *n* (%) for categorical variables; ^¥^ Results for Kruskal–Wallis test with multiple comparisons, significance is considered based on *p* values for Bonferroni correction for multiple comparisons; SO = sarcopenic obesity; NSO = non sarcopenic obesity; NSNO = non sarcopenic non obesity; ^a,b^ values with different superscripts are significantly different at *p* < 0.05; CHO = carbohydrates; kcal = calories; PUFA = polyunsaturated fat; MUFA = monounsaturated fat; ^†^ Fiber cut off point: daily intake < 28 g/day or ≥28 g/day.

**Table 3:** Logistic regression analysis for the association between protein intake (g/kg/day) and SO (*n* = 216).

Variable	Simple Univariate Model ^1^					Adjusted Multivariate Model ^2^				
	Coefficient	SE	Wald	*p*-Value	OR (95%CI)	Coefficient	SE	Wald	*p*-Value	OR (95%CI)
Age (years)	0.076	0.19	16.14	<0.001	1.08 (1.04–1.12)	0.050	0.02	6.324	0.012	1.051 (1.011–1.093)
Protein g/kg/day	−3.919	0.725	29.184	<0.001	0.020 (0.005–0.082)	−4.082	0.877	21.681	<0.001	0.017 (0.003–0.094)
Caloric intake kcal/day	−0.001	3.70 × 10^−4^	6.962	0.008	0.999 (0.998–1.00)	0.001	4.97 × 10^−4^	1.627	0.202	1.001 (1.000–1.002)
Fibers g/1000 kcal										
Fibers < 28 g/day					1.00					1.00
Fibers ≥ 28 g/day	−1.517	0.623	5.920	0.015	0.219 (0.065–0.745)	−1.215	0.667	3.316	0.069	0.297 (0.08–1.097)

^1^ Simple logistic regression model for each variable; ^2^ model including age, energy-standardized protein intake, and fiber intake; SO = sarcopenic obesity; SE = Standard error; OR = Odds ratio, 95%CI = 95% Confidence interval of the OR.

**Table 4:** Performance of the “g protein per kg” cut-off points to screen for SO (*n* = 216).

Model	*n*	AUC	*p* Value	Cut-Off Point	Sensitivity	Specificity	Specificity at 90% Sensitivity	Sensitivity at 90% Specificity
Simple model	45/216 (20.8%)	0.8049	<0.0001	0.99	0.73	0.73	0.45	0.42
Age-adjusted model	45/216 (20.8%)	0.8149	<0.0001	0.98	0.71	0.70	0.51	0.53

## Data Availability

The original contributions presented in this study are included in the article. Further inquiries can be directed to the corresponding author.
